# Modified Powder-in-Tube Technique Based on the Consolidation Processing of Powder Materials for Fabricating Specialty Optical Fibers

**DOI:** 10.3390/ma7086045

**Published:** 2014-08-22

**Authors:** Jean-Louis Auguste, Georges Humbert, Stéphanie Leparmentier, Maryna Kudinova, Pierre-Olivier Martin, Gaëlle Delaizir, Kay Schuster, Doris Litzkendorf

**Affiliations:** 1 Xlim Research Institute, UMR 7252 CNRS/University of Limoges, 123 avenue Albert THOMAS, 87060 LIMOGES, France; E-Mails: humbert@xlim.fr (G.H.); stephanie.leparmentier@xlim.fr (S.L.); kudinova@xlim.fr (M.K.); pierre-olivier.martin@unilim.fr (P.-O.M.); 2 SPCTS, UMR 7315 CNRS/University of Limoges, European Ceramic Center, 12 rue Atlantis, 87068 LIMOGES, France; E-Mail: gaelle.delaizir@unilim.fr; 3 Leibnitz Institute of Photonic Technology IPHT, Albert-Einstein-Str. 9, 07745 Jena, Germany; E-Mails: kay.schuster@ipht-jena.de (K.S.); doris.litzkendorf@ipht-jena.de (D.L.)

**Keywords:** powder in tube, process, glass, metal, oxidation state, multimaterial optical fiber, species diffusion, specialty optical fiber

## Abstract

The objective of this paper is to demonstrate the interest of a consolidation process associated with the powder-in-tube technique in order to fabricate a long length of specialty optical fibers. This so-called Modified Powder-in-Tube (MPIT) process is very flexible and paves the way to multimaterial optical fiber fabrications with different core and cladding glassy materials. Another feature of this technique lies in the sintering of the preform under reducing or oxidizing atmosphere. The fabrication of such optical fibers implies different constraints that we have to deal with, namely chemical species diffusion or mechanical stress due to the mismatches between thermal expansion coefficients and working temperatures of the fiber materials. This paper focuses on preliminary results obtained with a lanthano-aluminosilicate glass used as the core material for the fabrication of all-glass fibers or specialty Photonic Crystal Fibers (PCFs). To complete the panel of original microstructures now available by the MPIT technique, we also present several optical fibers in which metallic particles or microwires are included into a silica-based matrix.

## 1. Introduction

Standard silica singlemode optical fibers are widely used in many applications, such as optical components, fiber sensors, non-linear optical devices, and of course, for long-haul telecommunications.In the 1970s, research efforts were mainly focused on the development of efficient processes for elaborating highly transparent materials and for fabricating optical fibers with attenuation coefficient as low as possible in the telecom wavelength bands.

Chemical Vapor Deposition (CVD) techniques and synthesized-silica glass became the main actors of optical fiber success, with an attenuation coefficient record as low as 0.15 dB/km at the 1550 nm wavelength (PureBand^®^ Sumitomo, Japan). In the 1990s, rare-earth doped silica based optical fiber was an important breakthrough, offering the ability to amplify light without any optic/electronic converter and to develop optical fiber lasers.

Another breakthrough occurred in 1996 [1,2,3] with the introduction of the concept of Photonic Crystal Fiber, which is composed of a silica-core surrounded by a two dimensional (2D) periodic pattern of air-holes (running along the fiber length). The following decade was mainly devoted to the development of original PCF topologies for specific applications. For example, highly dispersive PCF at the telecom band (with chromatic dispersion lower than −1000 ps/(nm.km) at 1550 nm) [4] were presented as corrective optical devices for long-length data transmission applications. All-solid or hollow-core PCFs were also studied as active media for generating non-linear effects or as passive components for gas sensors, for example.

The slump in the demand on the telecommunication market in the last 15 years has initiated the use of optical fibers in other application domains such as optical sensors, optical fiber lasers, nonlinear devices, and high power delivery cables, requiring shorter fiber lengths (often limited to a few meters). The constraint on the optical losses has then been released, providing access to the use of other optical glasses with specific properties (e.g., higher linear and nonlinear refractive indices) but with a larger attenuation coefficient (than a pure silica one). New fiber fabrication techniques have also been proposed or adapted from previous ones. For example, the core suction method that consists in sucking up molten lead-SF6 glass (Schott) into a silica tube for realizing the fiber preform enables the fabrication of short length of optical fiber with soft-glass materials (glasses with lower transition point temperature (T_G_) than silica) [5]. A similar process was developed for inserting (along few centimeters) soft-glass or metal into an optical fiber by applying an external pressure on molten materials for filling the air holes of a PCF [6].

Others groups in the academic world have also worked on optical fibers composed of multimaterials. Ballato and colleagues (at Clemson University, United States) have reused the rod-in-tube technique for fabricating specialty optical fibers from a rod of a specific material (such as Silicon) filled in a silica tube [7]. They have also used the powder-in-tube method that consists in filling a tube with a powder material and drawing directly this preform down to an optical fiber [8]. This method, developed in the 1970s [9], appears to be a nice and simple way to produce optical fibers composed of multimaterials. Recently, Silitec Fibers SA proposed and patented [10] an extension of this concept for producing single-mode optical fibers by replacing the thick and expensive overcladding silica tube of the fiber preform (fabricated by MCVD) with synthetic pure silica powder, leading to a large reduction of the fabrication costs.

This industrial development was achieved by adding an intermediate process stage that consists in consolidating the preform filled with powder, for enabling stable and long fiber length productions with high reproducibility. A few years later (in 2007), we started to work at the Xlim research institute on the development of multimaterials optical fibers. In this context, in strong collaboration with Silitec Fibers SA, we have developed an original consolidation unit for improving the fabrication of multimaterials optical fibers with the powder-in-tube technique. The principles of operations of this Modified Powder-in-Tube (MPIT) technique, its advantages and fabrication constraints, are presented in Section 2. Examples of specialty optical fibers fabricated with MPIT technique are presented in Section 3 in order to demonstrate its potentialities for developing optical fibers composed of different glasses or of silica and metal.

## 2. The Powder Consolidation Process

### 2.1. Description of the Fabrication Process

In the last 20 years, the use of powders in optical fiber preforms has demonstrated interesting results for drawing state-of-the-art specialty optical fibers. Glass powders can be used to form the core and/or the optical cladding of the preform. Powder based processes present several advantages compared to conventional techniques like MCVD, extrusion or rod-in-tube techniques. It offers large freedoms in the fiber designs allowing the realizations of symmetric or asymmetric fiber topologies, composed of two or more different material compositions, each material inserted somewhere into the fiber structure. Furthermore, it can be easily combined with the stack-and-draw process [1] enabling the association of the properties of powder materials with PCF ones. Most of all, it allows for the fabrication of optical fibers from a large variety of optical materials (glasses, ceramics, metals) reduced to powders, in contrast with CVD techniques that are limited by the availability of gas precursors and to their low doping concentrations into silica glass.

Even though this fabrication process can be easily described—basically, it consists in drawing down to fiber (with a diameter of few hundred of micrometers) a glass tube filled with powder(s)—all the phenomena involved during the fabrication are complex and difficult to anticipate theoretically. An important phenomenon to manage during the drawing stage is the air trapped between the powder particles. During the high temperature processing like the preform drawing stage, air could expand suddenly causing severe perturbations such as formation of uncontrolled air holes into the fiber core or cladding, leading to large propagation losses, or as in the worst case, mechanical break of the fiber during its fabrication.

The size distribution and packing density (solid filling ratio in the porous material) of powder particles are parameters of great importance that determine the air fraction in the powdered preform. In order to reduce the porosity of the powder-based preforms, we propose a compaction heat-treatment of the preform under low pressure at a temperature above the transition temperature (T_G_) of the glass powder material.

**Figure 1:**
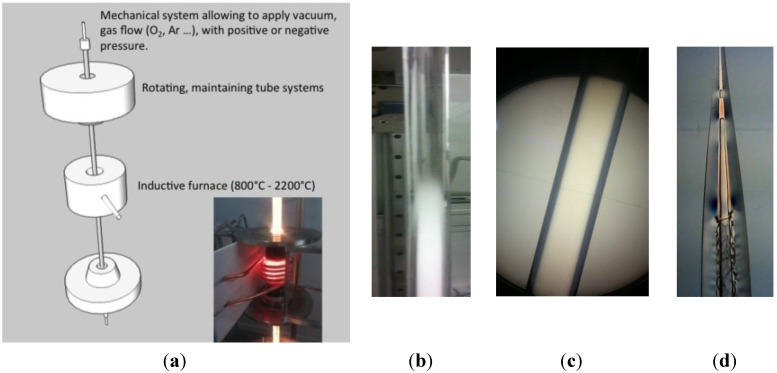
(**a**) Scheme of the Consolidation Unit; (**b**) photograph picture of a pure silica powder vitrified only in the half top. Pictures of pure silica powder based preform under observation with a polariscope; (**c**) after vitrification process showing absence of large defects and mechanical stress; (**d**) without consolidation (remaining part after the fiber drawing stage) showing cracks and large air inclusions in the core.

- For T_G_ < T < T_SOFT_ (with T_SOFT_ the glass softening temperature), the glass grains initiate a molten state and are able to deform. Primary vacuum ensures slight air elimination and granulates tend to agglomerate. After cooling, the final aspect of the preform is a spongious-like solid rod in the surrounding glass tube. This process is called preform consolidation.
- For T_SOFT_ < T, the glass powder material is softening and air can evacuate by natural bubbles collapsing that is enhanced by vacuum pumping. After cooling, the preform is a dense glassy rod. This process is called preform vitrification (see Figure 1b,c).

In this way, we are able to produce all-solid preforms from powdered raw materials without formation of air bubbles or cracks during the drawing stage.

Such processes are realized by using specific equipment named “Consolidation Unit”, developed at the XLIM Research Institute in collaboration with the Silitec Fibers SA company, Boudry, Switzerland (Figure 1a). In this equipment, both ends of the preform are hermetically sealed and connected to a vacuum pump or gas mass-flows for pressurizing it with helium or oxygen gas. Gases pressures or depressions are either controlled from the top or the bottom of the preform. The preform is held vertically with rotary mandrels. Preform rotation is applied to large diameter preforms for keeping homogeneous consolidation processing in the whole preform cross section. An inductive furnace (with a heating zone of 7 cm) is moved up and down along the preform. All parameters of the consolidation process are controlled by dedicated software.

In conclusion, the powder-in-tube technique could be significantly improved by a consolidation processing since it enables (i) a vitrification of the glass powder for removing air trapped between particles and for allowing stable, reproducible fiber drawings; (ii) the use of specific gases or gas mixtures during the preform heat-treatment for cleaning or for oxidizing or reducing materials into the perform; (iii) the realization of new fiber designs by implementing rods and tubes anywhere in the material powder (with or without a triangular arrangement).

### 2.2. Processing Features

#### 2.2.1. Thermo-Mechanical Property Mismatches between Fiber Materials

Optical fibers are made of at least with two different core/cladding materials. That the thermo-mechanical properties are mismatched between these two materials is an important characteristic to take into account for the consolidation process and the fiber drawing stage. Indeed, it may involve formation of large mechanical stresses at the fiber core/cladding interface leading to large optical losses, uncontrolled birefringence and mechanical fiber brittleness. This phenomenon is lowered by using materials with a similar thermal expansion coefficient (α) and liquidus (for crystals) or transition (for glasses) temperatures.

The thermo-mechanical stress (σ) induced in an optical fiber composed of disparate core and cladding glass materials could be estimated at a temperature T with the following Equation [11]:

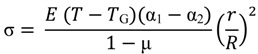

where the indices 1 and 2 correspond respectively to the fiber core and cladding, μ and *E* are the Poisson ratio and Young’s modulus of the glass in the core, and *r*/*R* is the core/fiber radius ratio. The ultimate compressive strength of pure silica is 1.1 GPa, *i.e.*, silica breaks when a force higher than this value is applied on it. Additional stresses on the fiber structure may also appear during the drawing process, due to technological defects or glass quenching.

Therefore, the consolidation process of glass powder preforms (before the drawing stage) is suitable for fabricating optical fibers composed of multimaterials with different thermo-mechanical properties:

- In the case of a low T_G_ mismatch between the glass core and pure silica cladding (T_G_(SiO_2_) ~ 1200 °C), the consolidation of the powder leads to rearrangements of macroscopic defects (reduction of the air fraction, annealing of glass powder materials…).
- In the case of a large T_G_ mismatch between the glass core and pure silica cladding, the consolidation process leads to an all-solid preform by softening only the core glass material.

To test and demonstrate the interest of the consolidation process we have fabricated several multimaterial optical fibers with different thermo-mechanical properties. These fibers were fabricated by filling a silica tube with different glass powders.

**Figure 2:**
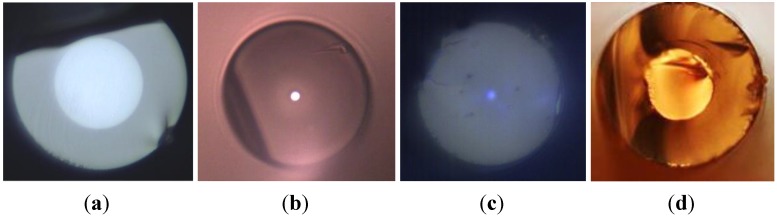
Microscope pictures of multimaterial optical fibers realized by the Modified Powder-in-Tube (MPIT) technique with (**a**) large SAL glass core and pure silica cladding; (**b**) lead-silicate glass core and pure silica cladding; (**c**) a borosilicate glass core surrounded by pure silica cladding; (**d**) Tellurite core material and borosilicate cladding.

A lanthano-aluminosilicate (SAL) glass developed at the IPHT Institute in Jena, (Germany), with the following composition: 82% SiO_2_-11.6% Al_2_O_3_-6.4% La_2_O_3_ was first used as the core material [12]. This glass presents a T_G_ ~ 900 °C and α ~ 2.8 × 10^−6^ K^−1^ (7 times higher than pure silica). Tens of meters of fiber long length have been successfully fabricated (Figure 2a). An optical fiber with a core composed of a lead-silicate optical glass, for which T_G_ ~ 600 °C (half compared to silica T_G_) and α ~ 10^−5^ K^−1^ (20 times higher than pure silica) has also been fabricated (Figure 2b). Figure 2c shows the cross section of fiber composed of borosilicate core (T_g_~ 560 °C) and a silica cladding.

To emphasis further the suitability of the MPIT technology for fabricating specialty optical fibers composed of glasses with different thermo-mechanical properties, we have realized a fiber composed of a tellurite glass core and a borosilicate cladding. Borosilicate is used to reduce the thermo-mechanical mismatch induced by the tellurite glass T_g_ (350 °C). Using borosilicate tubes for the preform preparation is a suitable way for fabricating optical fibers in which the core material has an important optical interest but presents a very low T_G_ compared to pure silica.

#### 2.2.2. Thermal Consolidation under Controlled Reducing or Oxidizing Atmospheres

The consolidation process enables to monitor air or gas pressure during the process. This is also useful to initiate redox chemical reactions into the preform structure. The oxidation state of materials can be controlled and changed during the heat-treatment process.

We have exploited this functionality for realizing specialty optical fibers with metallic palladium (Pd) particles embedded into the silica glass cladding [13]. Such optical fibers are difficult to fabricate with more conventional techniques such as rod-in-tube or CVD methods, since they are limited by the metal concentration range and the materials or precursors availability.

**Figure 3:**
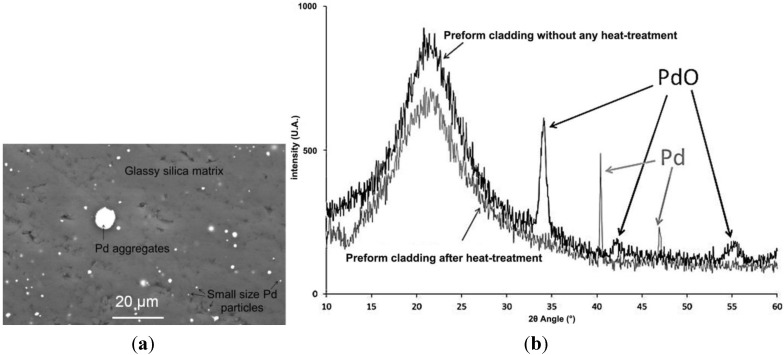
(**a**) SEM observation of the cross-section of a preform after heat-treatment, observation in the SiO_2_-Pd cladding with initially 1% mol PdO; (**b**) comparison of XRD diagrams for non-heat-treated and heat-treated 99% SiO_2_ + 1% PdO (% mol) composite materials (the large pic centered to 2θ = 20°–22° is due to the silica amorphous network and is typical to all glass structures).

Preforms were realized by using pure SiO_2_ and PdO powders mixture for the cladding material. The vitrification stage was performed under primary vacuum at a temperature close to the silica softening temperature. Scanning Electron Microscopy (SEM) observations (*Cf.* Figure 3a) coupled with EDS elemental analysis (Energy Dispersive X-ray Spectroscopy) and XRD (X-Ray Diffraction) measurements (BRUKER D8 equipment, Bruker, Rheinstetten, Germany) (*Cf.* Figure 3b) on fabricated samples demonstrate total reduction of the palladium oxide (Pd^2+^) to metallic palladium particles (Pd^0^) into the silica cladding material during the preform consolidation process.

Palladium aggregates formations are observed on SEM pictures in the silica cladding. Nevertheless, the mean metallic particles diameter was estimated (from the XRD diagram by using the Debye-Scherrer approximation) to be 60 and 40 nm (with some aggregates as shown in Figure 3a) for initial PdO concentration of 1% mol or 0.5% mol in the silica powder, respectively. The size of the metal particles can be influenced by the initial PdO concentration as well as the dwell time and temperature of the thermal reduction process.

#### 2.2.3. Chemical Diffusion between the Core/Cladding Materials

An important effect that can occur during the preform consolidation and the fiber drawing is the diffusion of species.

As an example, hydrogen diffusion through silica has been intensively investigated due to its negative impact on optical losses in the telecom bands or its positive effect for increasing the germanium-doped core sensitivity to UV laser excitation (for Bragg grating inscription) [14]. Diffusion effects have also been observed in optical fiber with high GeO_2_ doped silica core [15,16]. As species diffusion occurs along their concentration gradient, generating in the molten zone of the preform during the drawing process diffusion, from the core to the cladding, while oxygen and silicon atoms migrate from the cladding to the core. Spontaneous diffusion of phosphorus from the cladding to the core has also been demonstrated, even during the preform preparation by the MCVD method.

Diffusion of species has also been reported in optical fibers fabricated by the rod-in-tube techniques, *i.e.*, in fibers with much larger concentration gradients. As an example, during the realization of crystalline-core/glass-cladding optical fibers [17], the crystallinity of pure or doped YAG (Yttrium Aluminium Garnet) rods was used as the core material vanished during the fiber drawing stage. The authors have also noticed the influence of the fiber core diameter on the diffusion magnitude, demonstrating that smaller core diameter leads to larger diffusion of silicon (*i.e.*, silica) from the cladding to the core.

It is therefore essential to study chemical diffusion phenomenon in multimaterial optical fibers fabricated by the MPIT technique. Optical fibers composed of a SAL core and silica cladding have been fabricated with different core diameters for investigating species diffusion.

The refractive index profiles (RIP) of the fabricated optical fibers were measured by using an EXFO NR-9200 Optical Fiber Analyzer (Quebec, QC, Canada, based on the refractive near field technique) working at the 667.94 nm wavelength, with a spatial resolution of 0.5 μm and a measurement accuracy of 10^−4^.

**Figure 4:**
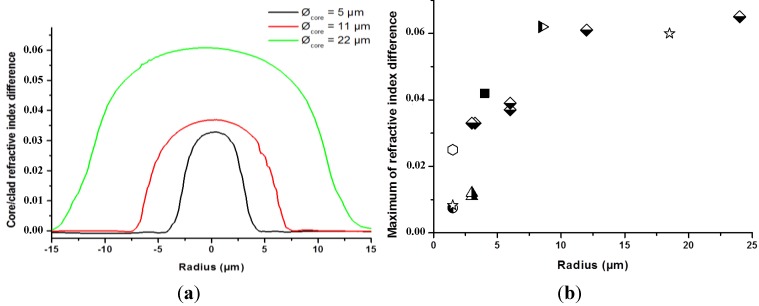
(**a**) Refractive index profiles for some SAL/silica fibers with different core diameters but realized during the same drawing run; (**b**) influence of the SAL core radius on the maximum of the core/cladding refractive index difference. Different forms of points correspond to different drawing campaigns.

For each fiber, the refractive index profile of the core presents a gradient shape, although the preform has a step-index shape indicating that migration of species has occurred during the drawing stage. One preform was drawn down to different diameters for investigating the core diameter influence on the diffusion phenomenon. As expected, the amplitude of the refractive index gradient decreases with the fiber core diameter, as shown in Figure 4a,b illustrates the maximum of the core/cladding refractive index difference value *versus* the core radius for all the optical fibers fabricated from different preforms. These results emphasize and confirm that smaller core leads to lower amplitude of the core/cladding refractive index gradient.

**Figure 5:**
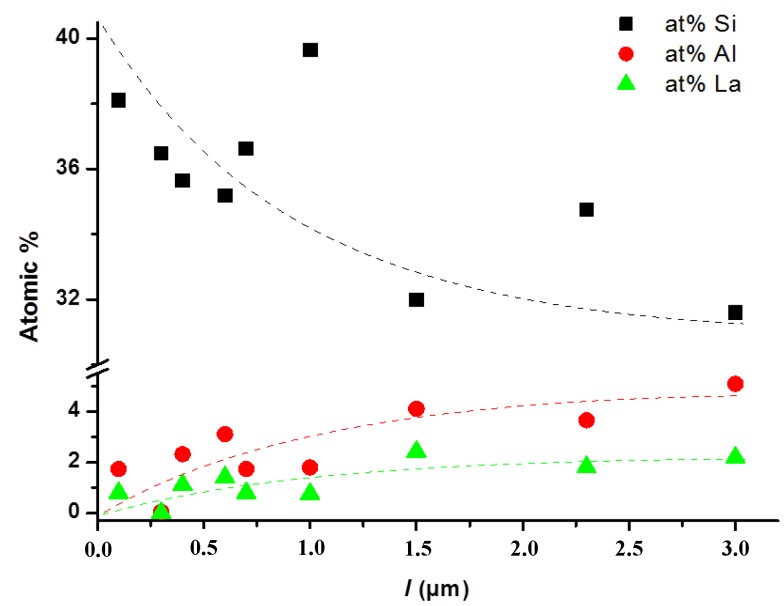
Atomic concentration of silicon, aluminum and lanthanum measured in a SAL core *versus* the distance (*l* = *r_core_* − *r*) from the silica-cladding/SAL-core interface.

In order to understand the origin of the gradient index shape of the SAL/silica fibers RIP, atomic concentrations of silicon, aluminum and lanthanum were investigated by EDS elemental analysis measurements on several fibers (with a core diameter of 6 μm or smaller). Measurements have been realized at different locations on the cross section of the fiber core. Figure 5 presents the atomic concentrations plotted *versus* the distance (*l*) from the cladding/core interface, *i.e.*, *l* = 0 at the core/cladding interface and *l* = 3 μm at the fiber core center. These measurements clearly demonstrate the evolution of the Si, Al and La atomic concentrations in agreement with the gradient shape of the RIP of the optical fiber. Lanthanum and aluminum concentrations are larger at the core center than at the core-cladding interface leading to a higher refractive index value.

Furthermore, the initial atomic concentrations of the Si, Al and La species in the SAL glass used are 27.33%, 4.64% and 2.56%, respectively. It is worth noting that measured atomic concentration of Si is much larger than the theoretical one. This value is around 39% at the core-cladding interface and decreases to 32% in the core center. This behavior could be explained by the diffusion of silicon from the silica cladding to the SAL glass core. This is in agreement with reported observations on optical fibers fabricated by the rod-in-tube technique presented in [17]. In contrast, diffusion phenomena from the core to the cladding material are observed for lanthanum and aluminum by the reduction of their atomic concentration (but below theoretical values) at positions closer to the core-cladding interface. These behaviors are induced by the concentration gradient between the SAL glass core and the pure silica cladding. They also explained the shape of the RIP and the reduction of refractive index magnitude.

This study demonstrates that the diffusion of species in the fiber core and cladding materials are important phenomena which ones have to be considered for designing and fabricating specialty optical fibers by the MPIT technique.

## 3. Examples of Specialty Optical Fibers Fabricated with the Modified Powder in Tube Technique

### 3.1. Lanthano-Aluminosilicate Glass Core/Silica Cladding Optical Fibers

This process is extremely well adapted for fabricating optical fibers composed of a core and a cladding with different optical materials. Typically, the cladding is formed by the tube (silica or borosilicate glass) and the core is realized by filling the tube with the selected optical material under the powder form.

Researchers at IPHT Institute in Jena (Germany) have intensively developed compositions in the SiO_2_-Al_2_O_3_-La_2_O_3_ glass system (*i.e.*, SAL glasses) [12]. Lanthana is an interesting component for increasing linear and nonlinear refractive indices of glasses. Alumina is used to ensure lanthanum solubility into the vitreous matrix. Among several glass compositions studied, the composition 82%SiO_2_-11.6%Al_2_O_3_-6.4%La_2_O_3_ has been used for fabricating optical fibers composed of a SAL core and a silica cladding. The linear and nonlinear refractive indices of this SAL glass are respectively *n* = 1.545 (at the 580 nm wavelength) and *n*_2_ = 5 × 10^−2^^0^ m^2^/W (at the 1064 nm wavelength) allowing large core/cladding refractive index difference (Δ*n* = +8.6 × 10^−2^) and enhanced nonlinear effects compared to pure silica.

**Figure 6:**
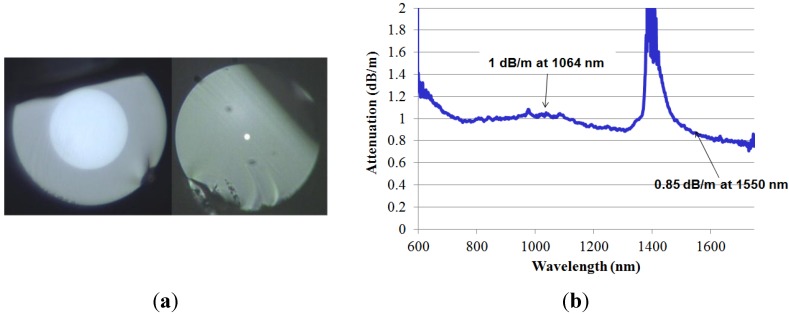
(**a**) Microscope pictures of cross section of two of SAL-core/silica-cladding optical fibers realized by the MPIT process ((**left**): core/fiber diameters = 50/95 μm; (**right**): 2.5/110 μm); (**b**) Attenuation coefficient *versus* wavelength of a SAL-core/silica-cladding optical fiber with a core diameter of 2.5 μm (measured by the cut-back method with fiber sample lengths of 28 and 5 m).

Several optical fibers have been fabricated with this SAL glass. After cleaning SAL glass powder and the surrounding silica tube, the powder is inserted into the tube and the preform is consolidated by heating it at a temperature between SAL glass T_G_ and T_SOFT_. Silica tubes with different inner diameters have been used to fabricate optical fibers with different core diameters enabling multimode or singlemode guidance in the near infrared spectrum. Cross section pictures of optical fibers with large and small SAL glass cores are shown in Figure 6a.

**Figure 7:**
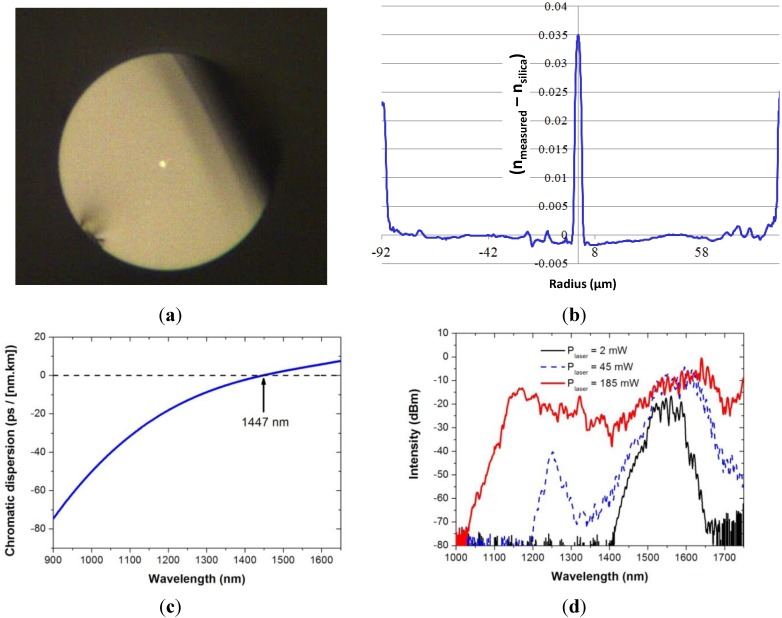
(**a**) Microscope picture of the cross section of an optical fiber composed of a SAL core of 3.7 μm and a silica cladding (with an external diameter of 210 μm); (**b**) RIP of the fiber (measured at 66,794 nm with EXFO NR9200); (**c**) chromatic dispersion *versus* wavelength of the fundamental mode guided in the fiber core. Measurements were done on a fiber sample of 40 cm long length (with a low-coherence interferometric setup in Mach-Zehnder configuration); (**d**) transmitted spectra of femtosecond pulses (centered around 1.55 μm) propagated in the fiber (sample length = 3 m) with different input powers.

It is worth noting that the bulk material absorption of SAL around 0.6 dB/m at the 1.2 μm wavelength (measured at the IPHT Institute) leads to an attenuation coefficient of the fabricated optical fiber around 1 dB/m in the near infrared spectrum (1000–1600 nm), as shown in Figure 6b. Even if this attenuation coefficient is large, the larger linear refractive index (+8.6 × 10^−2^) and nonlinear refractive index compared to silica ones are interesting properties for developing functionalized or component optical fibers. For example, we have fabricated a specific step-index SAL optical fiber for generating nonlinear effects with a laser that emits femtosecond pulses around the wavelength of 1.55 μm (*Cf.* Figure 7a). The fiber was fabricated with a small core diameter (3.7 μm) in order to take advantage of the large core-cladding step index for tailoring the chromatic dispersion curve of the fundamental mode and for enhancing nonlinearities (*i.e.*, increasing the nonlinear coefficient). As shown on the measured refractive index profile of the fiber plotted in Figure 7b, the step-index difference is around 3.5 × 10^−2^. This value is lower than predicted (Δ*n* = +8.6 × 10^−2^) due to silica diffusion from the cladding to the fiber core (*Cf.* Section 2.2). The chromatic dispersion of this SAL glass composition is equal to zero around 1791 nm. The diffusion of silica associated with the strong confining of the light within a small core allow to shift this zero dispersion wavelength (ZDW) down to 1447 nm (*Cf.* Figure 7c), enabling generation of nonlinear effects in anomalous dispersion regime by using pump laser operating around 1.55 μm. This example demonstrates that even simple fiber design could lead to interesting results when the core is made of a specific material.

In this prospect, a 3 m long length sample of this optical fiber was pumped with a laser emitting femtosecond pulses around 1.55 μm (with pulse width <100 fs, average power ~200 mW and repetition rate = 110 MHz). The generated nonlinear effects lead to a large spectral broadening of the laser output spectrum as shown in Figure 7d. More details on this experiment can be found in [12].

### 3.2. Multi Glasses Microstructured Optical Fibers

This process is also extremely well adapted for fabricating microstructured optical fibers, *i.e.*, fibers with more complex topologies by associating it with others fabrication processes such as the stack-and-draw technique. This method is the most used way for fabricating PCF. It consists in stacking glass capillaries and/or rods into a silica tube that is drawn down to structured glass rods (canes) of few millimeter diameters. The PCF is finally fabricated by drawing down a glass tube filled with a cane to a fiber of few hundred micrometers diameter. This technique offers much more flexibility for the fiber design, providing access to new properties and functionalities. The salient properties of PCF are endless single mode propagation, tailored chromatic dispersion curve, extremely high nonlinear coefficient and/or high birefringence.

**Figure 8:**
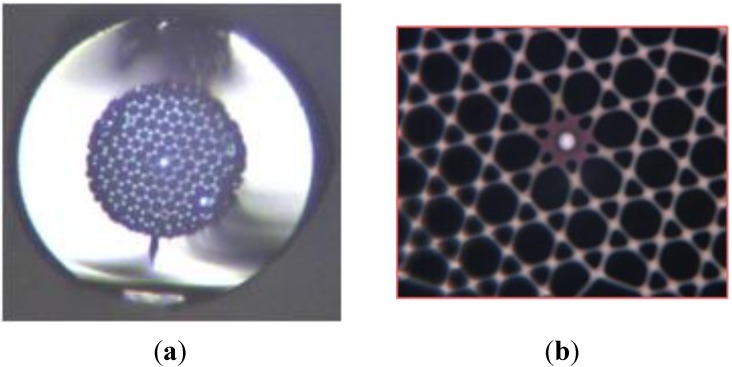
Microscope pictures of a PCF composed of a SAL core with a diameter of 1.25 μm (**a**) whole fiber cross-section (**b**) zoom in the core.

To demonstrate the possibility of associating both fabrication processes for taking advantages of PCF technology and material optical properties, we have realized a PCF composed of a SAL glass core. The fiber was simply performed by replacing in the stack, the silica rod used to form the core by a fabricated rod composed of SAL glass surrounded by a thin silica layer (result of the silica tube used in the MPIT process). A picture of the fiber cross section is shown in Figure 8a. The core with a diameter of 5.6 μm is composed of a 1.25 μm SAL glass core surrounded by a silica layer. The microstructured cladding of air holes induces strong light confinement leading to a large nonlinear coefficient and to a shift of the ZDW down to shorter wavelength enabling generation of nonlinear effects as reported [18] in similar SAL glass based PCF fabricated with an alternative process (the SAL glass core rod was machined after the glass casting and fine cooling into a parallelepiped mold).

The stack-and-draw technique is also used for fabricating another class of PCF that guides light into a hollow-core or solid-core by photonic bandgap effects. Solid-core Photonic BandGap (PBG) fibers are composed of a silica core surrounded by a 2D crystal of rods with a higher refractive index [19]. The light guidance is driven by this crystal cladding which one exhibits bandgaps in specific guiding conditions, leading to transmission windows with unusual optical properties.

**Figure 9:**
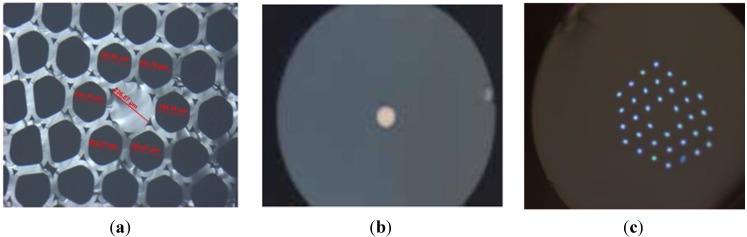
(**a**) Photograph picture of the PCF cane filled with SAL-core/silica-cladding optical fibers; (**b**) microscope picture of the cross section of the SAL-core/silica-cladding optical fiber (external diameter = 150 μm); (**c**) microscope picture of the fabricated fiber composed of a silica core surrounded by a 2D pattern of SAL glass inclusions.

In order to demonstrate the potential of the consolidation process of powder materials as an alternative solution for developing special fibers, we have fabricated a solid-core PBG fiber composed of a 2D crystal composed of SAL glass rods surrounding a silica core. The fiber was fabricated by filling the air holes of a PCF cane (composed of a silica core as shown in Figure 9a) with SAL-core/silica-cladding optical fibers (*cf.* Figure 9b). The PBG fiber is then fabricated by drawing down a silica tube filled with the cane. A cross section picture of the solid-core PBG is shown in Figure 9c. The fiber is composed of a 2D crystal made of two rings of SAL glass rods with a diameter of 2 μm spaced by 7.5 μm.

It is worth noting that this fiber could also be realized by drawing (in two steps) a stack of SAL glass and silica rods. This fabrication demonstrates the interest of the MPIT process combined to the stack-and-draw technique for developing solid-core PBG fibers or related ones. In contrast with usual solid-core PBG fibers that are based on Ge-doped rods fabricated by MCVD process, this original technological route offers more flexibility in the choice of the rod materials and as a consequence on the conception of the fiber properties. For example, using rods with a larger refractive index compared to SAL glass or silica ones may increase the light confinement into the core leading to lower bend loss sensitivity and to a modification of the chromatic dispersion curve.

### 3.3. Metal–Silica Optical Fibers

The flexibility of the MPIT process allows us to use other materials than pure glass compositions. Metals could also offer interesting properties and functionalities to optical fibers. To demonstrate the potentiality of the MPIT technique for fabricating new specialty fibers composed of metals and glasses, we have investigated and tested the realization of three classes of fibers composed of (i) a silica core including copper particles; (ii) a copper wire surrounded by a silica cladding; (iii) a PCF with copper wires into the air/silica microstructure.

#### 3.3.1. Optical Fibers with Metallic Ions and Particles Embedded into the Core

Metallic nanoparticles (e.g., Au, Cu, Ag) have unique optical and electrical properties that differ, especially optical absorption, drastically from bulk material. The physical origin of the light absorption by the metallic nanoparticles is the excitation of coherent oscillations of the conduction band electrons by light that is known as surface plasmon resonance [20]. This phenomenon has been employed to optical switches, waveguides, spectroscopy, chemical and biological sensors, *etc.*, [21,22,23,24]. As a consequence, metallic nanoparticles embedded and stabilized into optical fibers pave the way to many types of applications with such a simple device.

Optical fibers with copper ions into the core present interesting properties, as extremely high resonant nonlinearity coefficient exhibited by Cu^2+^ (*i.e.*, CuO) ions for example [25]. Moreover, modification of the copper ions oxidation states from Cu^2+^ (*i.e.*, CuO) to Cu^+^ (*i.e.*, Cu_2_O) under gamma radiations are measurable regarding the luminescence spectrum of the copper, allowing the development of gamma ray dosimeter [26].

Such optical fibers could be prepared by inserting metallic oxide during the realization of the preform with CVD techniques for example. Flash vaporization process could also be joined to the MCVD method to allow silica layers doping with many heavy metal or rare earth-ions that do not have precursor materials in gaseous form compatible with CVD techniques [27]. However, it is worth noting that these processes enable only the insertion of metallic ions through metallic oxide precursors, since to the best of our knowledge, common CVD processes do not allow for working on reducing atmosphere. Furthermore, these processes require preform collapsing operated at very high temperature and under depression. This fabrication stage is critical for particles embedded in the latest deposited layers which ones can be removed during this operation.

**Figure 10:**
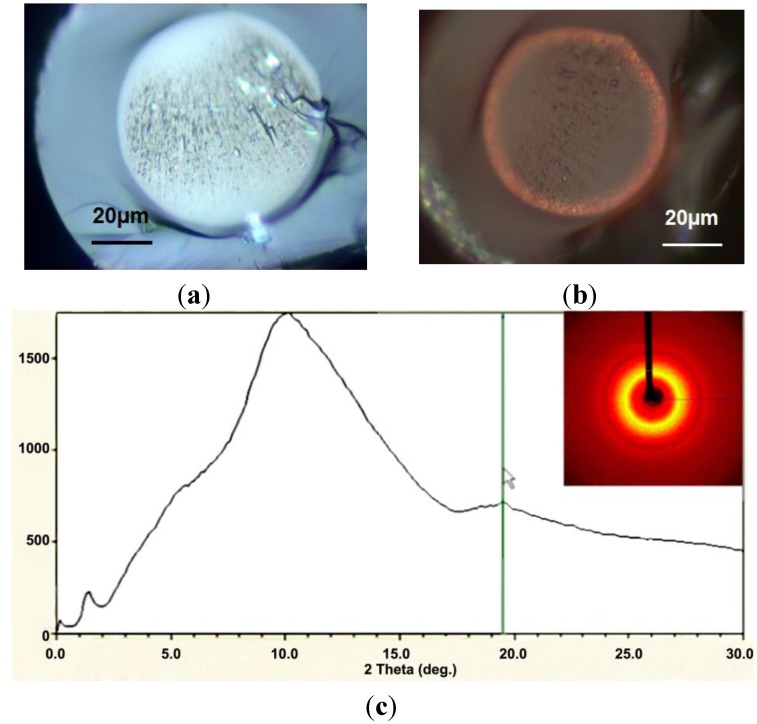
Microscope picture of cross section of a SAL glass +0.5% CuO core optical fiber **(a)** before and **(b)** after a post-drawing heat-treatment at 850 °C under a reducing Ar-H_2_ (5%) atmosphere (**c**) X-Ray Diffraction (XRD) pattern of the SAL-Cu optical fiber (Kα-Mo, Kappa CCD).

Others techniques, such as the simple powder-in-tube technique are also willing to produce metallic particles embedded into optical fiber. First trials have been performed using a silica cladding and a core made of 70% SiO_2_-20% Al_2_O_3_-10% La_2_O_3_ (SAL’) + 0.5% CuO (mol%) (*Cf.* Figure 10a). A post drawing heat-treatment at 850 °C (>T_G_) under a reducing Ar-H_2_ (5%) atmosphere leads to the presence of a highly red reflective ring at the interface core-cladding as shown in Figure 10b. XRD pattern of the core fiber (*Cf.* Figure 10c) was obtained using a Single Crystal X-Ray Diffraction System Kappa-CCD (Kα-Mo = 0.71 Å). We clearly show a broad peak centered at 2θ = 10° corresponding to the glassy core and a small peak at 2θ = 19.5° highlighted by a green line corresponding to nanometric copper particles Cu^0^. The formation of this Cu^0^ ring at the interface core/cladding results from the reduction of Cu^2+^ and Cu^+^ ions due to the Ar-H_2_ atmosphere in the fiber after drawing. To balance the depleted charge that results from the reduction reaction at the interface core/cladding, Cu^+^ and Cu^2+^ ions diffuse from the core center to the interface and are reduced also leading to the ring. It is noteworthy that the heat-treatment near T_G_ (850 °C) allows ions to become mobile.

As demonstrated in Section 2.2.2, the MPIT process can be directly used to reduce in one step Pd^2+^ into Pd^0^ under vacuumed conditions. By regarding the Ellingham’s diagram of some metal/metal oxide redox couples, this technique could be extended also to other metallic particles than palladium, including copper. Different trials are still in progress.

#### 3.3.2. Long Metallic Wires Distributed in Optical Fibers

**Figure 11:**
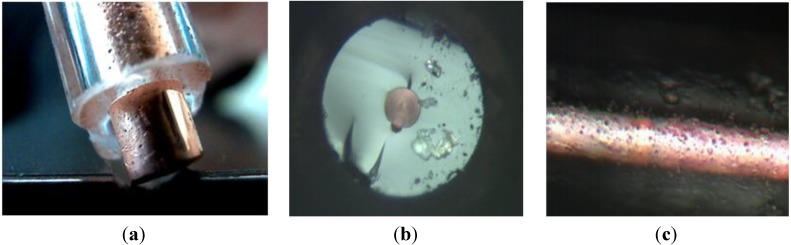
(**a**) Photograph picture of a silica preform with a pure copper core. Microscope pictures of the fabricated fiber with a copper-core diameter of 50 μm; **(b)** cross section; (**c**) longitudinal picture.

Another possibility offered by the MPIT process is the fabrication of optical fibers including metallic wires. To explore this potentiality, we have started by fabricating a fiber composed of a copper wire surrounded by a silica cladding. A silica tube filled with pure copper powder was consolidated and then drawn down to fiber with an external diameter of 200 μm. As shown in Figure 11b, the fiber is composed of a copper wire with a diameter of 50 μm surrounded by a silica cladding. It is worth noting that, even if there is a large difference between the softening temperature of silica (1665 °C) and the fusion temperature of copper (1080 °C), we were able to draw hundreds of meters of such fibers.

In order to quantify the quality of this copper wire, its resistivity was measured on a fiber sample of two meters long. The evaluated resistivity is 1.7 × 10^−8^ Ω·m, which is very close to the theoretical one (1.68 × 10^−8^ Ω·m).

This realization demonstrates the interest of the MPIT technique for developing high quality electric micro-wires (in diameters) for specific electrical or RF applications. It also gives us basic skills and expertise for developing specialty optical fibers that associate optical and metallic wire properties.

**Figure 12:**
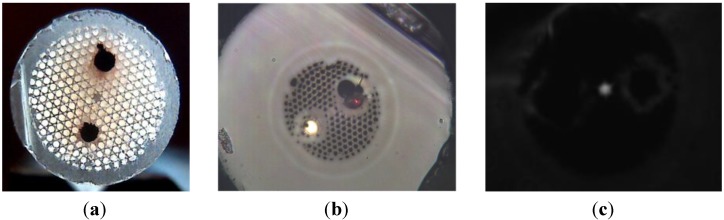
(**a**) Photograph picture of a fabricated PCF cane with two copper rods; (**b**) microscope picture of the fabricated PCF with two copper wires of 20 μm diameters; (**c**) measured near-field optical pattern of the light guided in the fiber core.

In this respect, we fabricated a PCF with copper wires inserted into the air/silica microstructured cladding. To fabricate it, we inserted two larger capillaries in the PCF stack that were filled with consolidated copper rods. This stack was drawn down to cane of few millimeter diameters (*Cf.* Figure 12a) and then a silica tube filled with a cane was drawn to a fiber of 250 μm diameter. A picture of the fiber cross section composed of a silica core surrounded by a photonic crystal cladding of air holes and two copper wires of 20 μm diameters, is shown in Figure 12b. Light propagation in the core (along few centimeter long length fiber sample) was successfully measured with a CCD camera, as shown by the near-field picture of the fundamental mode in Figure 12c.

Even if, the drawing process could be improved this first fabrication result demonstrates the high potential of the MPIT technique for developing specialty optical fibers with added functionalities.

## 4. Conclusions

We have presented in this paper an original route to produce specialty optical fibers, based on the powder-in-tube technique combined with a consolidation process realized under specific oxidizing or reducing atmosphere.

After the presentation of the principle of operation of this process for consolidating or vitrifying a powder based optical preform, we demonstrated its flexibility for drawing materials with large thermo-mechanical different properties, for controlling and modifying oxidation states of powder materials inside the preform. We also investigated experimentally the chemical diffusions of materials in the preform (during the fiber fabrication) and demonstrated their impacts on the fiber RIP and optical properties.

We presented preliminary fabrication results of optical fibers composed silica and a lanthano-aluminosilicate glass used as the core material in step-index fiber or in specialty PCFs. To complete the panel of original microstructured fibers now available by the MPIT technique, we also presented several optical fibers in which metallic particles or microwires are included in a silica-based matrix.

We believe that we have demonstrated in this paper the flexibility and simplicity of the MPIT technique compared to classical manufacturing processes, in order to develop new class of specialty optical fibers.
